# Effect of Educational Program Based On Exercise Therapy on Burned Hand Function

**Published:** 2014-01

**Authors:** Fatemeh Mohaddes Ardebili, Zahra Sadat Manzari, Mehri Bozorgnejad

**Affiliations:** 1 Medico-Surgical Group, School of Nursing and Midwifery, Iran University of Medical Sciences, Tehran, Iran;; 2 Medico-Surgical Group, School of Nursing and Midwifery, Mashhad University of Medical Sciences, Mashhad, Iran

**Keywords:** Hand Burn, Hand Function, Education, Burn Patients, Exercise Therapy

## Abstract

**BACKGROUND:**

Hands burn was associated with significant functional disorders that severely affected patient’s quality of life. The aim of this study was to examine the effect of educational program based on exercise therapy on burned hand function.

**METHODS:**

This experimental research was conducted in a period of ten months in 2010-2011 in Mottahari Hospital in Tehran in Iran. The sample included 60 patients, who were randomly assigned into experimental and control groups, half in intervention and half in control group. Educational program was implemented on experimental group. The data collection tools were two observational checklists about determining of hand function.

**RESULTS:**

Both groups were matched in characteristic of demographic and burn injury. Subjects in experimental group demonstrated significant improvements in range of motion and hand function balance from admission to discharge.

**CONCLUSION:**

In order to reduce the hand functional impairment caused by burns, it is recommended that special attention be paid to patient education about exercise therapy.

## INTRODUCTION

Hand impairment is a potential and considerable factor in chronic disabilities because it causes complications such as deformation and functional disorders such as difficulty of normal movement and disorder of hand normal movement^1^ which will have negative effect on life quality of the patients.^2^^,^^3^ A study has shown that 36% of body injuries related to hands and 39% of burn injuries also included hands and some parts of elbow. Involvement of hands following burn has been reported 72% in some studies and 85% in electrical burns.^4^^,^^5^ Very important issue in hand burns is proper management of hands treatment and care for maximizing normal function of burned hand and on this basis, rehabilitation of hands is an essential principle in effective care of patients.^6^^,^^7^ In burned hands rehabilitation, it is compulsory to encourage the patients to move in range of motion. Unfortunately, this main issue is not considered in most patients and its consequences are shown after a while as all kinds of deformation and functional disorders.^7^ In fact, incomplete rehabilitation or delayed rehabilitation is an important factor for impairing function of hands and negative consequences resulting from it.^4^ A study found that most patients with hand burns even with partial degree had some degrees of scar, range of motion (ROM) and cantracture.^5^ Due to widespread and permanent complications of burn , burn impairmnt nature is different from that of other impairments,^8^^,^^9^ therfore, it requires careful treatment than other impairments.^10^^,^^11^ Different studies have shown that the patients who received proper care did not show effects of scar or deformation; in addition, these patients are able to return to work and perform routine work and also quality of life is not affected after burn.^12^^,^^13^ In all of these studies, education to patient has been emphasized as a strong and effective lever for preventing deformation and scars in patients care process.^14^^-^^17^

In fact, the most important point in physical therapy is to have a proper educational program which is easy to understand for the patients.^18^^-^^20^ Although education to patient has positive effect on clinical improvement trend and complications caused by burn, studies show that those who take care of patients particularly nurses do not pay enough attention to physical therapy education for the burned hands and this issue plays effective role in functional disorders and handicaps caused by it.^21^ On the other hand, optimal and effective execution of motions is considered even in case of physical therapy training to patient and asking him to perform it properly and insufficient cooperation and lack of motivation in patient are regarded as the justifiable factors of the educational programs inefficiency. However, if patient and consequences of physical therapy are regarded important and he understands importance of physiotherapy, his cooperation will increase.^20^ Considering importance of self-care educational program and also considering that most patients do not receive sufficient and effective education according to experiences of researcher and results of studies and consequently, they suffer from the complications of burn, therefore, researchers decided to study effect of education on function of burned hands by formulating physical therapy educational program among patients with hands burn. General goal of this study was to determine effect of physical therapy educational program on function of hand among patients with hand burn hospitalized in Shahid Mottahari Hospital, Tehran, Iran. 

## MATERIALS AND METHODS

This research was a clinical trial. The intervention factor was hand care education and had intervention and control groups. The control group was the one which received physiotherapy routine educations in Shahid Mottahari Hospital, Tehran, Iran and the intervention group received the designed physiotherapy educational program. At the start of the study, all samples were selected with convenience sampling considering inclusion criteria and then each one of them was assigned a number. By selecting numbers randomly, the samples were included in two intervention and control groups (each containing 30 persons). 

The research population included all patients with hand burn and the research samples were all patients with second degree burn and third degree burn on surface of hands with burn degree of 15-45%. They were hospitalized in hospital at time of research and suffered from burn for 72 hours. They had age of between 15 and 65 years and were able to talk and establish communication and could read and write. Exclusion criteria included affliction with disorders such as diabetes mellitus (due to probability of having motor and sensory neuropathy), dermal allergy, malignancy, record of mental diseases, deformation and motor disorders of hands and fingers. Two intervention and control groups were equal in terms of controllable variables including age, literacy, marital status, job, residence, burn factor, cause of burn, burn site, burn surface and also type of supportive system of hospitalization ward. 

In addition, uncontrollable variables of the study included personal characteristics of the samples in following educational program which was not controllable in this research and were regarded as limitations of the research. Research hypothesis was that hand function in patients who received the designed educational program was different from the patients who did not receive this program. To determine the necessary sample size in each one of two intervention and control groups according to view and estimation of the esteemed statistics expert, the confidence level was 95% and statistical power was 90% showing that 80% of the patients with hand burn were discharged from the hospital with normal dysfunction, in case the designed education could reduce this rate by 40%, the education was regarded effective and 30 persons were estimated in each group using the related formula for determining sample size. 

The data collection tools were two observational checklists. In addition, two questionnaires including demographic information and burn wound information questionnaire were used for studying the patients. On the first day of selecting each sample, they were completed for all control and intervention group samples. Comparison of normal function of hands in two control and test groups after education based on Jebson’s 7-item scale and also observational checklist for determining range of motion were measured. Jebson’s scale was an objective and standardized tool which was designed and described by Jebson *et al*. in 1969.^22^

In addition, reliability of the tool was determined between 0.7 and 0.95 in different studies.^23^^,^^24^ In these instruments, normal function of hands was measured with 7 hand movement assessment objective criterion including ability to write a 24-letter sentence, eating (simulated feeding with a spoon and five kidney beans), ability to lift objects (such as glass), turning objects (5x3 card such as visit cards), arranging small objects (such as paper clips, bottle plug or coin), arranging large light objects (such as arranging large cubes : coffee cans or canned tuna) and arranging heavy objects of 1 pond beside each other (such as ability to displace large glasses filled with water or tomato paste cans (about 0.5 kg) and putting them beside each other)^18^ and after translating it from Persian, its content validity was confirmed by the corrected opinions of 10 specialists and professors and their reliability was confirmed by intra-observer agreement test (k=0.87). These instruments were measured for all patients in intervention and control groups at time of discharging the sample first for dominant hand and then for non-dominant hand. In the above checklist, score 1 was the only capturing with hand, score 2 with help of both hands, score 3 was complete performance.

To prevent bias, all questionnaires were filled by a physiotherapist colleague of researcher. In addition, he was not informed of his inclusion in control or intervention group. To observe ethical notes, a booklet and educational CD were given to him when each patient in control group was discharged. In the intervention group, the researcher executed the designed educational program for physiotherapy. Educational program was first executed individually for each patient in the first 72 hours after burning. In this program, all educational needs for taking care of hands such as importance, necessity and effects of physical movements of hands, prevention from deformation and functional disorders, importance of cooperation of patient in physiotherapy were considered. 

Then, the patients were informed of the cases available in the program. In other words, a group educational session was held as group discussion after completing individual educational sessions. In this session, they were trained about necessity and performance of exercises and physiotherapies in hands. In addition to speech and group discussion, visual means such as film, photo and slide were used. At the end of each educational session, all patients were asked to inform the researcher of all ambiguities and questions. In addition, educational contents were reviewed through question and answer method. Arrangement was made with physician and physiotherapist about performance of joints and wrists movements, time and number of physiotherapies. To ensure exercises, the researcher directly observed patients of both groups for exercises. Telephone number of the researcher was given to all patients in intervention and control group to answer their questions. Term of each educational session was 30-45 min, the number of individual educational session was 1 to 2 sessions and the number of group educational session was 2-3 dependent on need of patients which was considered, controlled and recorded in each educational session. Average term of hospitalization of both groups was 1 month. Comparison of disorder in normal function of hands between two test and control groups specified result of the research. Training place was considered with coordination of hospital’s manager in each ward. The data was analyzed using SPSS software (version 12, Chicago, IL, USA). Ethical proposal was confirmed by ethical committee of Iran University of Medical Sciences. 

## RESULTS

In this study, totally 60 patients were studied. Thirty patients were included in the control group and 30 patients in the intervention group. Most patients in the control group were male (60%), single (51.7%), aged between 15 and 25 years (46.7%), held primary school degree (46.7%), resided in village (53.6%), had weak condition (income of lower than 80 US$ per month) (56.7%) and supportive system including family, spouse and relatives (16.6%). In addition, the reason for burn was self immolation (70%), burn site was head and neck, face, body and hands (63.3%) and burn factor was petrol (37.9%) with total burn level (36-45%). In the intervention group, most patients were male (56.7%), aged between 15 and 25 years (26.7%), married (63.3%), held primary school degree (43.3%) , resided in village (49.6%), had middle economic status (income of between 50 and 100 US$ per month) (43.3%) and supportive system including family, spouse and relatives (23.6%). In most patients in the intervention group, the reason for burn was self immolation (73.3%), burn site was head and neck, face, body and hands (53.3%), burn factor was petrol (34.5%) with total burn level (36-45%). Mean and standard deviation of patients’ age were 28.23 and 9.81 in the control group respectively and 31.43 and 8.91 in the intervention group. Independent t-tests, Chi Square, Mann-Whitney and Fisher Exact tests did not show statistically significant difference between two intervention and control groups in terms of demographic characteristics and also burn injury characteristics. Therefore, both groups were equal in terms of demographic characteristics and burn injury characteristics. It is necessary to note that all samples were right-handed and all had at least two burned hands (burn on the fingers to wrist). Mann-Whitney test showed ability to write with right hand, ability to lift objects (such as glass) (*P*=0.006) and with left hand, eating with spoon with right hand (*P*=0.001) and with left hand (*P*=0.002), ability to arrange small objects (plug of bottle) with right hand (*P*=0.000) and with left hand (*P*=0.034), ability to displace large light objects (tuna can) with right hand (*P*=0.006) and with left hand (*P*=0.01) and ability to arrange heavy objects weighing 1 pond with right hand (0.5 kg-tomato paste can) and also with left hand (*P*=0.002) between two control and intervention groups (Table 1). 

**Table 1:** Frequency distribution of burned patients in two test and control groups in terms of ability to perform seven functions of hand (Jebson’s scale).

**Group**		**Control ** **right hand**		**Intervention ** **right hand **		**Result of statistical test **	**Control ** **left hand**		**Intervention ** **Left hand **		**Result of Statistical test **
**Function and its determining cases**											
		**Number**	**Percent **	**Number **	**Percent **		**Number **	**Percent **	**Number **	**Percent **	
	Complete	0	0	12	40		0	0	0	0	
1-Ability to write (25 words)	With help of two hands	9	30	16	53.3	Mann-Whitney U= 261,000 P=0.03	10	33/3	10	33.3	Mann-Whitney U= 357,000 *P* value= 0.22
	Holding pencil in hand and illegible handwriting	6	20	2	6.7		0	0	4	13.3	
	Inability	15	50	0	0		20	7.66	16	4.53	
	Total	30	100	30	100		30	100	30	100	
2- Ability to lift objects (glass)	Complete	0	0	12	40	Mann-Whitney U= 285.500 P=0.006	0	0	7	3.23	Mann-Whitney U = 271.500 *P* value= 0.001
	With help of two hands	6	20	17	7.56		6	20	18	60	
	Only holding in hand	0	0	0	0		0	0	3	10	
	Inability	24	80	1	3.3		24	80	2	7.6	
	Total	30	100	30	100		30	100	30	100	
3- Ability to east with spoon	Complete	0	0	23	7.76	Mann-Whitney U= 223.500 P=0.0001	0	0	7	3.23	Mann-Whitney U = 223.500 *P* value= 0.002
	With help of two hands	11	7.36	6	20		8	7.26	17	7.56	
	Only holding spoon	0	0	1	3.3		0	0	1	3.3	
	Inability	19	3.63	0	0		22	3.73	5	7.16	
	Total	30	100	30	100		30	100	30	100	
4- Ability to arrange small objects (plugs of bottle)	Complete	0	0	25	3.83	Mann-Whitney U=247.000 P=0.0001	0	0	9	30	Mann-Whitney U = 327.000 *P* value= 0.034
	With help of two hands	10	3.33	4	13.3		9	30	15	50	
	Only holding in hand	0	0	0	0		0	0	3	10	
	Inability	20	7.66	1	3.3		21	70	3	10	
	Total	30	100	30	100		30	100	30	100	
5-Ability to displace light and large objects (cans)	Complete	0	0	11	36.7	Mann-Whitney U=280.500 P=0.006	0	0	5	16.7	Mann-Whitney U=295.000 *P* value= 0.010
	With help of two hands	0	0	16	53.3		0	0	17	56.7	
	Only holding in hand	0	0	1	3/3		0	0	6	20	
	Inability	30	100	2	6/7		30	100	2	6.7	
	Total	30	100	30	100		30	100	30	100	
6- Ability to arrange heavy objects on each other (tomato paste can)	Complete	0	0	18	60	Mann-Whitney U=261.000 P=0.002	0	0	8	7.26	Mann-Whitney U=276.000 *P* value= 0.002
	With help of two hands	11	7.36	12	40		8	7.26	18	60	
	Only holding in hand	1	3.3	0	0		0	0	4	3.13	
	Inability	18	60	0	0		22	3.73	0	0	
	Total	30	100	30	100		30	100	30	100	
7- Ability to turn objects (to turn card)	Complete	0	0	11	36.7	Mann-Whitney U=318.00 P=0.26	0	0	8	26.7	Mann-Whitney U=314.00 *P* value= 0.17
	With help of two hands	2	7.6	17	7.56		2	7.6	16	3.53	
	Only holding	0	0	1	3.3		0	0	3	10	
	Inability	28	93.3	1	3.3		28	93.3	3	10	
	Total	30	100	30	100		30	100	30	100	

On the other hand, Mann-Whitney statistical test did not show statistically significant difference between two control and intervention groups in terms of ability to turn objects (5 visit cards 5x3) with right hand (*P*=0.26) and with left hand (*P*=0.17) and also ability to write with left hand (*P*=0.22) (Table 1). In addition, to compare two groups in terms of motor ability of wrist and fingers of hands in range of motion, Chi Square test showed statistically significant difference between two groups in terms of ability to bend wrist of the right hand (*P*=0.001), ability to bend wrist of the left hand (*P*=0.019), rotate wrist of the right hand (*P*=0.005), ability to rotate wrist of the left hand (*P*=0.01) though there was statically significant difference between two control and intervention groups in terms of ability to extend fingers of the right hand (*P*=0.22) and left hand (*P*=0.55) and also ability to bend fingers of the right hand (*P*=0.100) (Table 2). 

**Table 2:** Frequency Distribution of burned patients in two test and control groups in terms of motor function in range of motion.

**Function **	**Group **	**Control ** **Right hand**		**Intervention ** **Right hand **		**Statistical test**	**Control ** **Left hand**		**Intervention ** **Left hand **		**Statistical test **
		**No.**	**%**	**No.**	**%**		**No.**	**%**	**No.**	**% **	
Ability to bend wrist	Yes	16	3.53	25	3.83	df=1 x2=5.31 *P=*0.013	13	3.43	25	3.83	df=1 x2=2.07 *P=*0.001
	No	14	7.46	5	67.16		17	7.56	5	67.16	
	Total	30	100	30	100		30	100	30	100	
Ability to rotate wrist	Yes	13	3.43	22	3.73	Chi=3.15 square *P=*0.019	9	30	20	67.66	Chi=5.85 square *P=*0.005
	No	17	7.56	8	67.26		21	70	10	33.33	
	Total	30	100	30	100		30	100	30	100	
Ability to bend fingers	Yes	13	3.43	7	3.23	df=1 X^2^=2.70 *P=*0.100	17	67.56	7	3.23	df=2 X^2^=8.62 *P=*0.01
	No	17	7.56	23	7.76		13	33.43	23	7.76	
	Total	30	100	30	100		30	100	30	100	

## DISCUSSION

Results of this research showed that execution of hands education program was effective on function of hands. Results of this study are in line with results of other studies though hand function measurement criterion is based on Jebsen’s Hand Function Test which may be different from the scales measured by other studies. For example, Flintham *et al.* (2005) in a study on 42 patients with burn showed that exercises were effective on improvement of joints movement and improvement of skin movement.^16^ Results of some studies show positive effect of physical movements education on prevention of all kinds of deformation and consequently improvement and preservation offhand normal function. Although out study has not dealt with creation of deformation which can have negative effect on hand function. For example, Petronic *et al.* (2005) in a three-year prospective study showed effect of premature physical movements on prevention from contracture, scar and kloid in patients with the second degree burn on hand.^17^ Study by Sabapathy *et al.* (2010) showed that education to patient for performig physiotherapy is one of the most important measures for managing deformations after hand burn.^18^ In addition, positive effect of physiotherapy on motor function of skin and also on application of all kinds of surgical interventions has been considered in some other studies but it was not studied in our study due to use of Jebsen’s scale. For example, Celis *et al. *(2003) in a clinical trial on 53 burned children showed that supervised exercises were effective on improvement of mobility and reduction of need for surgical interventions.^19^ In all of the above studies, the best strategies for performing physical exercises are proper education of these exercises to patients and also supervision on proper and effective performance of movements and both factors have been included in the designed educational program in the present study. Study by Kent *et al.* (2003) entitled study of effect of codified education on improvement of complications caused by silicon between 2001 and 2002 showed that codified care education (5 pages in writing and a video educational film for 6 months) was effective on quality and quantity of removing scar and kloid resulting from silicon and also improvement of normal function and hand motions in burned patients compared with common education in the control group.^14^ In this regard, results of study by Paratz *et al.* (2012) showed that physiotherapy educational program in patients with hand burn promoted physical and functional improvement and had positive effect on psychological consequences of these patients.^25^ O’Brien *et al.* (2012) also showed that execution of codified and founded educational programs based on educational need of patient increased obedience of patients with hand and upper limb burn to care and therapeutic diets.^26^ In line with results of this study, study by Zoubine *et al.* (2007) also showed that physiotherapy program in patients with hands burn considerably reduced complications of burning particularly contracture.^27^ Results of the present study, Schneider *et* *al.* (2011) showed that execution of rehabilitation programs particularly physiotherapy education in patients with hand burn had positive and considerable effect on range of motion of hands and normal function of hand. It is necessary to note that normal function of the burned hand was observed and measured using Jebson’s instrument in this study like the present study.^28^ Study by Grisbrook *et al.* (2012) which was conducted to study effect of exercise education on promotion of health and life quality of the deep burns survivors showed that physiotherapy education in patients with burns had positive effect on promotion of physical health level and also improvement of their life quality.^29^ Bodyw *et al.* (2010) also showed that massage in children with burn was effective on improvement of function and range of motion.^30^ Positive effect of physiotherapy education on hand function among the patients without burn has been also assessed. For example, Heine *et al.* (2012) in a widespread clinical trial on 1200 patients with rheumatoid arthritis showed that design of physiotherapy educational programs in patients with rheumatoid arthritis had positive effect on promotion of normal function of hand and also range of motion.^31^

It is necessary to note that ability to return objects in two control and intervention groups was not significant in the present study and considering inability to return objects completely in both groups at time of discharge should be reviewed and considered more in education of physiotherapy educational program for ability to return hand and also in education and track of movements by patients. In addition, results of the study are also applied by clinical nurses and physiotherapists in burned patients’ care planning in hospitals and burn centers. 

Since this study was conducted in limited time and focused on effect of physiotherapy education on hands burn and patients with special inclusion criteria and failure to follow patients after discharge (limitations of the study), therefore, it is advisable to conduct this study in burned patients by following them for 6 months to 1 year after burning and also evaluate effect of intervention of this study on function of other burned organs (feet) and also in burned patients with higher degrees of burn. 

Results of this study showed that physical exercise has positive effect on reduction of complications caused by burn. In fact, physiotherapy is very important in burned patient and directly prevents adherence, shortness of tissues and motor limitation of joints. One of the most important points in physiotherapy is to have proper educational program which can be understood by the patient. Therefore, considering results of this study and other similar studies, it is recommended to pay enough attention to codified care education for the burned organ in the hospitals with emphasis on exercises and continual supervision. 

## Footenote

Please cite this paper as:

Mohaddes Ardebili F, Manzari ZS, Bozorgnejad M. Effect of Educational Program Based On Exercise Therapy on Burned Hand Function. World J Plast Surg 2014;3(1):39-46.
